# Automatic segmentation and labeling of T1, T7, and T12 thoracic vertebrae in neonatal chest radiographs: a deep learning approach using nnU-Net framework

**DOI:** 10.3389/fped.2026.1673925

**Published:** 2026-02-05

**Authors:** Sumin Jung, Heerim Yun, Hye Won Cho, Jaeyoung Kim, Donghoon Yu, Jinho Son, Byung Min Choi

**Affiliations:** 1 Core Research & Development Center, Korea University Ansan Hospital, Ansan, Republic of Korea; 2 Coreline Soft Co. Ltd., Seoul, Republic of Korea; 3 Department of Pediatrics, College of Medicine, Korea University, Seoul, Republic of Korea; 4 Department of Convergence Medicine, College of Medicine, Korea University, Seoul, Republic of Korea; 5 Miso Information Technology Co. Ltd., Seoul, Republic of Korea

**Keywords:** automatic segmentation, chest radiographs, labeling, neonate, thoracic vertebrae

## Abstract

**Introduction:**

Identifying the thoracic vertebra visible on chest radiographs is a standard practice to assess proper position of a tube and catheter tips within their designated anatomical target regions in critically ill newborn infants. We introduce a fully automated deep learning system based on the nnU-Net architecture for segmenting and labeling T1, T7, and T12 in neonatal chest radiographs.

**Methods:**

We retrospectively collect 14,660 neonatal chest radiographs from 10 university hospitals in Korea, including both infants with tubes or catheters and those without. All images were deidentified and annotated for T1, T7, and T12 vertebrae using rectangular bounding boxes, validated by pediatricians. We split the dataset into training (11,860), validation (1,400), and test (1,400) sets, maintaining an even distribution by gestational age and birth weight.

**Results:**

The automatic segmentation algorithm demonstrated excellent agreement with human-annotated segmentation for the T1, T7 and T12 vertebrae [Dice similarity coefficient (DSC): 0.8327, 95% CI: 0.8237–0.8418; 0.8322, 95% CI: 0.8213–0.8432; 0.7998, 95% CI: 0.7864–0.8133, respectively]. To identify the approximate location of each vertebra, a relatively modest DSC threshold of 0.50 or 0.60 already yielded an accuracy above 90% for T1, T7, and T12.

**Conclusion:**

Our deep learning-based automated algorithm built on the nnU-Net framework could accurately segment and label T1, T7, and T12 thoracic vertebrae in neonatal chest radiographs. This artificial intelligence-driven approach can map anatomical target regions based on thoracic vertebrae for assessing the positioning of a tube and catheter tips in a neonatal intensive care unit.

## Introduction

An endotracheal tube and various intravascular catheters, such as umbilical artery and/or vein catheters, are commonly used in neonatal intensive care units (NICUs) for life-supporting purposes, especially in critically ill newborn infants. Tips of tubes and/or catheters should be placed in specific anatomic positions to ensure proper operation and to minimize the risk of related complications (1–3).

In neonates, identifying the thoracic vertebra visible on chest radiographs is a standard practice to assess proper position of a tube and catheter tips within their designated anatomical target regions (4). For example, the tip of an endotracheal tube should be placed between the first thoracic vertebra (T1) and the second thoracic vertebra (T2). The tip of an umbilical artery catheter for high position should be placed at T6–T9 and the tip of an umbilical vein catheter should be placed at T8–T10. Additionally, to determine an appropriate inhalation status in critically ill infants, the diaphragm's position is identified at the level of posterior ribs of thoracic vertebrae T7–T9.

Recent advances in deep learning for medical image analysis have enabled the development of various approaches demonstrating high accuracy in detecting and classifying tubes and catheters on neonatal chest radiographs (5, 6). However, the lack of research on anatomical target regions in chest radiographs for evaluating proper placement of a tube and catheter tips poses challenges for clinical application in neonates.

This study aimed to address this gap by providing information on anatomical target regions based on thoracic vertebrae in neonatal chest radiographs. To achieve this, we developed a fully automated deep learning system utilizing the nnU-Net architecture to segment and label T1, T7, and T12. The nnU-Net framework, a self-configuring deep learning segmentation system widely used for medical imaging, was selected to ensure robust and reproducible performance across diverse neonatal radiographs. By defining these vertebrae as consistent anatomical reference points, the proposed system seeks to facilitate accurate and objective evaluation of tube and catheter positioning in neonatal clinical practice.

## Methods

### Data collection and dataset division

We retrospectively collected 14,660 neonatal chest radiographs (October 2022–February 2023) from 11 university hospitals in Korea, including both infants with tubes or catheters and those without. This study was approved by the Institutional Review Board (IRB) of each participating hospital (approval no. 2022AS0056) with a waiver of informed consent.

All images were deidentified and annotated for T1, T7, and T12 vertebrae using quadrilateral polygonal labels to reflect vertebral orientation in rotated or tilted infants. Annotations were reviewed independently by two pediatricians and finalized by consensus. We split the dataset into training (11,860), validation (1,400), and test (1,400) sets at the patient level, ensuring balanced distributions of gestational age (GA) and birth weight (BW). The hospital-wise dataset composition, including GA and BW group distributions, is summarized in Table 1. To reduce identifiability, GA was categorized into three groups: <28 weeks, 28–32 weeks, and ≥33 weeks, and BW was also categorized into three groups: <1,000 g, 1,000–1,500 g, and ≥1,500 g. Additional steps, including the removal of patient identifiers and DICOM metadata, were taken to ensure anonymization.

**Table 1:** Dataset composition by hospital and demographics.

Hospital ID	No. of Images	GA01 (%)	GA02 (%)	GA03 (%)	BW01 (%)	BW02 (%)	BW03 (%)	With tube or catheter (%)
H01	1,700	72.5	7.5	20.0	73.3	6.8	19.9	69.4
H02	1,206	28.4	20.6	51.0	28.5	19.6	51.9	67.5
H03	1,974	36.8	24.8	38.4	33.5	17.3	49.2	64.7
H04	826	16.6	24.0	59.4	18.6	15.6	65.8	70.0
H05	1,384	14.9	12.9	72.2	15.1	11.5	73.4	72.6
H06	989	25.8	22.5	51.7	20.2	28.2	51.6	66.5
H07	443	49.0	40.9	10.2	31.0	65.2	3.8	62.7
H08	3,096	38.3	25.2	36.5	39.9	19.8	40.3	64.1
H09	1,815	59.6	26.6	13.8	56.8	30.9	12.3	70.1
H10	407	22.1	22.6	55.3	32.4	14.7	52.8	72.5
H11	820	93.7	1.3	5.0	22.6	16.0	61.5	67.6
Total	14,660							

Each pixel was labeled into five classes: background (0), lung (1), T1 (2), T7 (3), and T12 (4). This labeling provides a foundation for detailed segmentation and analysis of key structures involved in this study.

### Deep learning model

**Figure 1:**
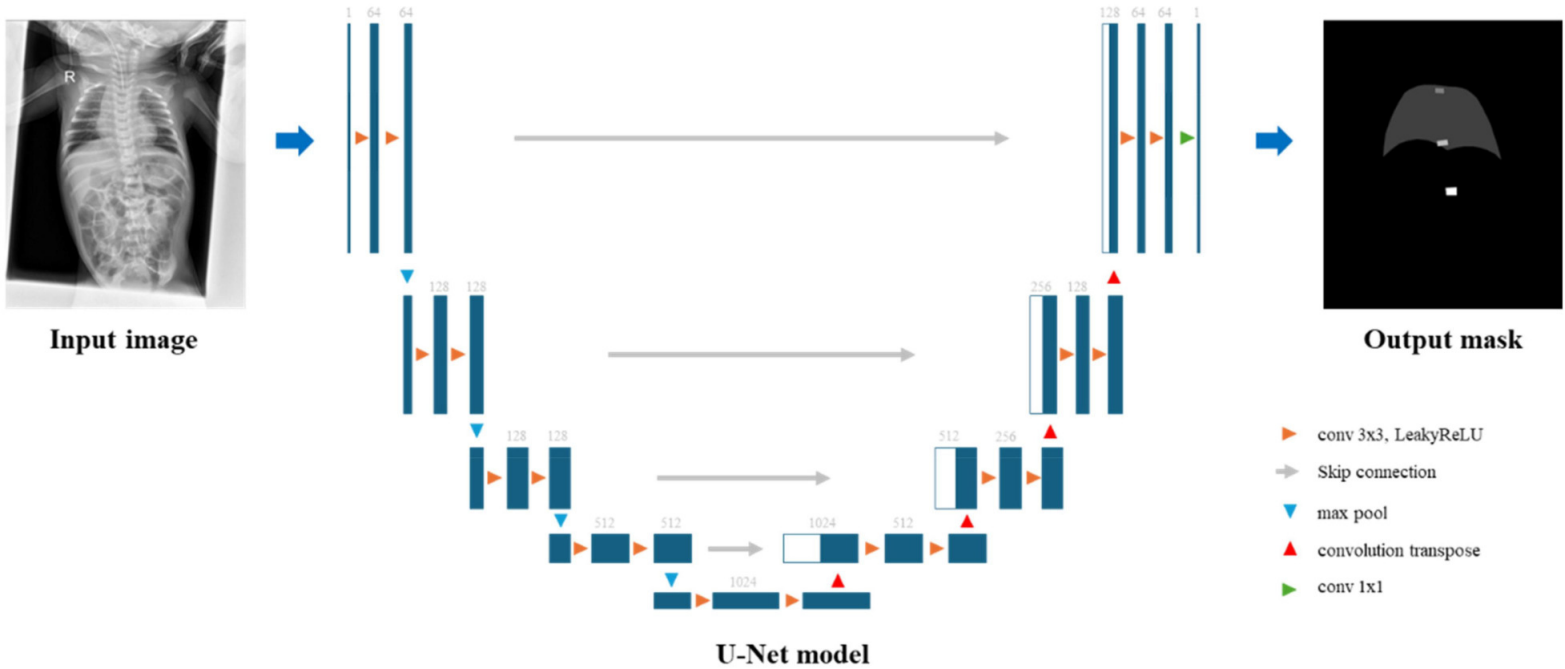
An overview of the proposed network architecture.

The nnU-Net framework, a two-dimensional U-Net architecture, was employed to segment T1, T7, and T12 vertebrae. The model runs on a high-performance workstation with GPU acceleration (7). Figure 1 shows an overview of the proposed network architecture. We used a batch size of 12 and a patch size of 448 × 576 pixels, leveraging nnU-Net's automated configuration for medical image segmentation. For training, we used a composite loss function combining Dice loss and Cross Entropy. The segmentation model was optimized using Stochastic Gradient Descent (SGD) with a Nesterov momentum of 0.99. A polynomial learning rate scheduler was used, starting with an initial learning rate of 0.01. The model was trained for 1,000 epochs to obtain final weights, and no early stopping was applied. In all experiments, a fixed random seed (102) was used to ensure reproducibility.

To support vertebral localization, the model was trained as a five-class segmentation task comprising background, lung, and the T1, T7, and T12 vertebrae. Lung was included as a contextual class, serving as a consistent anatomical landmark across variable neonatal postures and imaging fields, as illustrated in Supplementary Figure S1.

### Data augmentation

Various data augmentation techniques, including rotation, scaling, and noise injection, were applied to enhance the model's robustness and generalization. By introducing these transformations, the model could better handle diverse clinical scenarios without overfitting. Augmentation details are shown in Table 2.

**Table 2:** Segmentation performance as determined using various metrics.

Transformation	Parameter/Range (per image)	Probability	Description
Rotation	(−180 °, 180 °)	0.2	Rotate the image by a random angle within the specified range.
Scale	(0.7, 1.4)	0.2	Apply random zoom within the specified range.
GaussianBlurTransform	sigma = (0.5, 1.0)	0.2	Apply Gaussian blur with a randomly sampled sigma.
GaussianNoiseTransform	noise_variance = (0, 0.1)	0.1	Add Gaussian noise with a randomly sampled variance.
BrightnessMultiplicativeTransform	multiplier_range = (0.75, 1.25)	0.15	Scale pixel intensities by a random multiplier.
ContrastAugmentationTransform	contrast_range = (0.75, 1.25)	0.15	Adjust image contrast within the specified range.
SimulateLowResolutionTransform	zoom_range = (0.5, 1)	0.25	Downsample and upsample to simulate lower resolution
GammaTransform	gamma_range = (0.7, 1.5), invert_image = False	0.3	Apply gamma correction with a randomly sampled gamma; no inversion.
MirrorTransform	axis = (0, 1)	1	Mirroring along specified axes (In this work, x- and *y*-axis)

### Pre- and post-processing

We performed Z-score normalization and resized all images to have a uniform resolution. This ensured consistency across the dataset, allowing the model to focus on relevant features rather than variations in image intensity or scale.

Post-processing was performed using Connected Component Labeling (CCL) to eliminate spurious fragmented predictions and retain only the largest connected component for each vertebral class (T1, T7, T12). This step reduced noise and improved the anatomical plausibility of the segmented output, as shown in Supplementary Figure S2.

### Dice similarity coefficient (DSC)

To evaluate concordance between automated and manual segmentations, the Dice similarity coefficient (DSC) was primarily used. In addition, we computed Intersection-over-Union (IoU) values, 95% Hausdorff distances (HD95; mm), and mean surface distances (MSD; mm); all metrics are summarized in Table 3. Supplementary Figure S3 illustrates qualitative differences across DSC thresholds (0.3–0.8) and supports 0.5 as a practical cutoff for acceptable segmentation quality. It was calculated with the following formula: DSC = 2 (AS ∩ MS)/(AS + MS). DSC values ranged from 0 to 1. A DSC value of 0 indicated complete discordance between automated and manual segmentations, while a value of 1 signified perfect concordance and identical segmentation shapes (8). Because each thoracic vertebra (T1, T7, T12) was segmented as a separate class, we calculated DSC values for each class individually (DSC_T1, DSC_T7, DSC_T12). Additionally, since manual ground truth used quadrilateral polygon labels, reported DSC values might be conservative when predictions are smaller or differ in shape from annotated polygons. For patient-level reporting, image-level metrics were averaged per patient before summarizing.

**Table 3:** Overall segmentation performance at the patient level in neonatal chest radiographs, including dice similarity coefficients (DSCs), intersection-over-union (IoU) values, 95% hausdorff distances (HD95; mm), and mean surface distances (MSD; mm) for T1, T7, and T12.

Vertebra	DSC Mean ± SD	DSC 95% CI	IoU Mean ± SD	IoU 95% CI	HD95 Mean ± SD (mm)	HD95 95% CI (mm)	MSD Mean ± SD (mm)	MSD 95% CI (mm)
T1	0.8327 ± 0.1724	0.8237–0.8418	0.7384 ± 0.1731	0.7293–0.7475	8.4339 ± 8.2512	8.0017–8.8662	1.3720 ± 5.7211	1.0723–1.6717
T7	0.8322 ± 0.2094	0.8213–0.8432	0.7481 ± 0.2002	0.7376–0.7586	9.4905 ± 12.7707	8.8215–10.1595	2.3449 ± 9.5402	1.8452–2.8447
T12	0.7998 ± 0.2574	0.7864–0.8133	0.7174 ± 0.2401	0.7049–0.7300	13.2952 ± 18.0057	12.3520–14.2384	4.3954 ± 13.4379	3.6915–5.0993

## Results

### Training behavior and convergence

During training, both training and validation losses showed a steady decrease and eventually converged, indicating minimal risk of overfitting. Notably, the validation Dice Similarity Coefficient (DSC) converged within a range of 0.80–0.85 across T1, T7, and T12 vertebrae, mirroring the trend observed in the training set. This trend was consistent across hospitals with balanced distributions of gestational age (GA) and birth weight (BW) (Table 1), indicating stable training behavior across datasets. The dataset was balanced among gestational-age and birth-weight groups, which helped mitigate potential bias related to demographic variations. Validation and test performance remained stable between hospitals, demonstrating consistent model behavior across diverse clinical data.

Furthermore, the final performance on the test set closely matched validation results, with DSC values remaining within the same range, reinforcing the robustness of the trained model under real-world conditions. Learning curves showed stable convergence of training and validation losses, and validation DSC improved consistently without overfitting (Supplementary Figure S4).

### Quantitative segmentation performance

The performance of the segmentation model was evaluated using test datasets. Overall segmentation performance is summarized in Table 3, including DSC values (all >0.79), Intersection-over-Union (IoU) values, 95% Hausdorff distances (HD95; mm), and mean surface distances (MSD; mm), each reported with narrow confidence intervals across T1, T7, and T12. This accuracy would allow clinicians to identify key anatomical landmarks reliably.

### Threshold-based accuracy for clinical localization

To complement the primary evaluation, Table 4 presents accuracy rates at various DSC thresholds. For example, thresholds of ≥0.5 or ≥0.6 yielded over 90% accuracy for T1, T7, and T12, suggesting that the model can support clinical landmark identification even when perfect pixel-level alignment is unnecessary. A range of DSC threshold values (e.g., 0.50, 0.60, 0.70, etc.) was applied to determine whether each vertebra segmentation was acceptably accurate at the patient level. Given that our primary objective was to identify the approximate location of each vertebra, a relatively modest threshold of 0.50 or 0.60 already yielded an accuracy above 90% for T1, T7, and T12. This level of performance is generally sufficient for clinical tasks where exact pixel-level concordance is less critical.

**Table 4:** T1, T7, and T12 accuracies at the patient level in neonatal chest radiographs at different dice similarity coefficient (DSC) cutoff values.

Vertebra	DSC cutoff values					
	0.50	0.60	0.70	0.75	0.80	0.90
T1	0.9642	0.9571	0.9286	0.8907	0.8121	0.3393
T7	0.9443	0.9414	0.9321	0.9086	0.8607	0.4136
T12	0.9078	0.9064	0.8979	0.8807	0.8271	0.3721

### Qualitative assessment of segmentation

**Figure 2:**
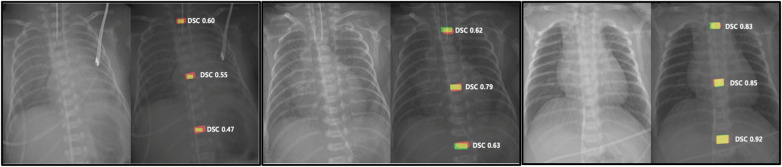
Representative visualization of segmentation results for three cases. Automatic segmentation results are shown in red. Manual segmentation ground truth is shown in green. Overlapping areas indicating concordance are shown in yellow. Dice Similarity Coefficients (DSCs) were used to quantify agreement between automated and manual segmentations.

To qualitatively evaluate the model's segmentation performance across various scales and cases, representative results are presented in Figure 2, where automatic predictions (red), manual ground truth (green), and overlapping areas (yellow) are visualized. Supplementary Figure S3 further illustrates qualitative differences across DSC thresholds (0.3–0.8), showing that DSC ≥ 0.5 corresponds to visually acceptable localization. Although DSC values varied, the automatic segmentation demonstrated strong visual concordance with the actual thoracic vertebral bodies, providing insight into model performance and potential directions for refinement.

## Discussion

### Overall clinical implications of vertebral labeling

To the best of our knowledge, this is the first study that demonstrates a fully automated deep-learning system designed to accurately segment and label thoracic vertebrae in neonatal chest radiographic images, particularly in assessing proper positioning of tube and catheter tips and the appropriate inhalation status.

In this study, the accuracy of our deep-learning system for labeling of each vertebra (T1, T7, T12) was evaluated using DSC. Patient-level accuracy based on various cutoff values of DSC is presented in Table 4.

In clinical applications, anatomical information on thoracic vertebrae in neonatal chest radiographs can be obtained using various DSC thresholds depending on the clinical purpose. Simply, for identifying and assigning numbers to the thoracic vertebra on neonatal chest radiographs, high accuracy can be achieved with a relatively low cutoff value such as 0.50.

However, in infants with very low birth weight, accurate identification of anatomical target regions is essential for assessing the proper positioning of tube and catheter tips. In these cases, precision is not only affected by the location, but also affected by the shape and size of the vertebrae. To achieve this level of precision, a higher cutoff value for the DSC is necessary, with 0.80 being clinically appropriate. Nonetheless, the algorithm used in this study demonstrated a high accuracy that would be clinically acceptable.

### Labeling strategy and multi-metric evaluation

During this study, trained experts performed manual segmentation of thoracic vertebrae. However, several limitations were encountered. Due to constraints of the segmentation tools, annotations were limited to rectangular regions. In addition, manual segmentation was also performed by several individuals using variously sized rectangular shapes. We found that automated segmentation was generally accurate, whereas manual results varied in consistency and precision, which lowered concordance and reduced DSC scores.

In our revised labeling strategy, vertebral regions were defined using quadrilateral polygons to better reflect vertebral orientation and reduce annotation–prediction shape mismatch. Accordingly, we reported standard segmentation metrics such as DSC, IoU with 95% confidence intervals, HD95, and MSD as primary outcomes (Table 3), while threshold-based accuracies (Table 4) provided a task-oriented supplementary view for clinical localization. Patient-level scores were calculated as per-patient averages of image-level metrics to support consistent interpretation at hospitals. These refinements collectively improved reproducibility and inter-hospital consistency.

Therefore, independent use of automated deep learning systems is expected to improve patient-level accuracy, with higher values observed at elevated DSC thresholds, demonstrating more precise concordance. The inclusion of a lung mask as an auxiliary input and the application of connected component labeling (CCL) during post-processing improved localization and reduced fragmented predictions (Supplementary Figures S1, S2), which suggests the system is reliable and well-suited for clinical use.

### Methodological considerations and limitations of the nnU-Net framework

Recent advancements in digital image analysis utilizing deep learning have positioned artificial intelligence as an indispensable tool in the medical field. Specifically, medical image classification has proven valuable in aiding healthcare professionals to make more accurate clinical decisions and reduce the risk of misdiagnosis (9, 10).

In recent years, deep convolutional neural networks (CNNs) have demonstrated exceptional efficacy in various pattern recognition tasks, including object detection, semantic segmentation, and classification in medical imaging, underscoring their potential to revolutionize the analysis and interpretation of medical images (11, 12). Deep classification methods in particular are well-suited for the proposed task, as these models require only image-level labels for training, thereby minimizing the need for detailed annotations (13). In particular, utilizing architectures such as nnU-Net allows for robust performance for performing medical image segmentation tasks due to its self-configuring nature and adaptability to various datasets (7).

In our study, the nnU-Net framework offered remarkable performance for thoracic vertebra segmentation. However, it primarily focuses on pixel-level segmentation tasks without directly integrating anatomical priors or clinical workflows. This limitation means that while the model can accurately delineate shapes of the vertebrae, it could not fully account for their anatomical context or nuances of clinical decision-making. In future studies, incorporating rule-based approaches or additional imaging modalities (e.g., CT or MRI) could enhance the model's ability to capture spatial relationships and clinical significance of segmented structures.

### Future directions for clinical translation

Clinically, our study facilitated precise determination of the thoracic vertebrae's location and size in neonatal chest radiographs, laying the groundwork for additional applications. Based on these findings, we aim to develop an integrated algorithm that not only can segment vertebrae, but also can identify adjacent anatomical regions to verify positions of tubes or catheter tips. Such an algorithm would be particularly beneficial for extremely low birth weight infants, in whom even minor deviations in tube or catheter placement can have significant clinical implications. Extending this approach to include other relevant structures (e.g., ribs, lung fields) could further augment computer-aided diagnostic systems, ultimately improving the safety and effectiveness of NICU interventions.

In conclusion, we propose a fully automated deep learning-based algorithm built on the nnU-Net architecture and designed to accurately segment and label thoracic vertebrae in neonatal chest radiographs. This artificial intelligence-driven approach can map anatomical target regions based on thoracic vertebrae for positioning tubes and catheter tips.

In future prospective studies, deep learning algorithms for multi-class classification of neonatal tubes and catheters should be integrated with these anatomical target regions based on thoracic vertebrae. Incorporating this approach into computer-aided diagnostic systems in the NICU may enhance the accuracy of tube and catheter tip localization, thus enabling neonatologists to perform more timely and precise assessments in clinical practice.

## Data Availability

The original contributions presented in the study are included in the article/Supplementary Material, further inquiries can be directed to the corresponding author.
