# Supplementary material for: Automatic segmentation and labeling of T1, T7, and T12 thoracic vertebrae in neonatal chest radiographs: a deep learning approach using nnU-Net framework
Source: Front Pediatr. 2026 Feb 5;14:1673925. doi: 10.3389/fped.2026.1673925 (PMC12916647; doi:10.3389/fped.2026.1673925)
Supplement: Supplementary file 1 [file Datasheet1.pdf]

**<Supplementary Figures>**

(A)

(B)

(C)

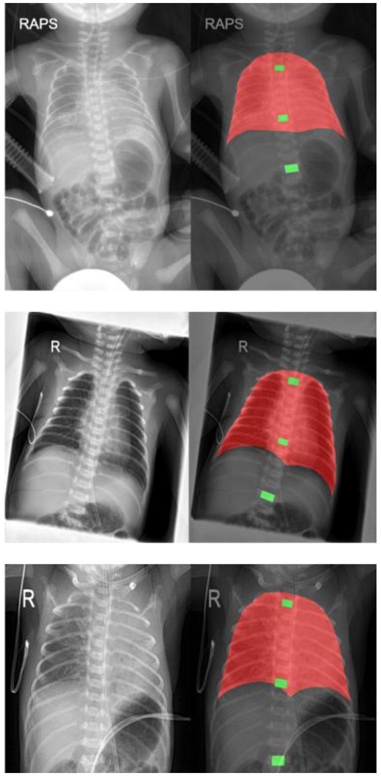


**Supplementary Figure 1. Inclusion of a lung mask (auxiliary channel) to guide vertebral localization.**

**The semi-transparent lung region (red) serves as an anatomical landmark for various neonatal postures and imaging conditions.**

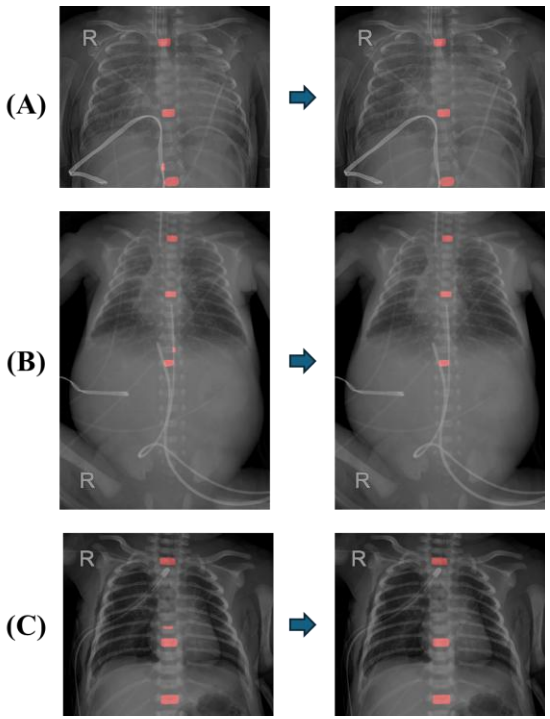


**Supplementary Figure 2. Post-processing using connected component labeling (CCL).**

**For each vertebral class, the largest connected component is retained to eliminate spatially insignificant fragments and refine the final mask.**

(A)

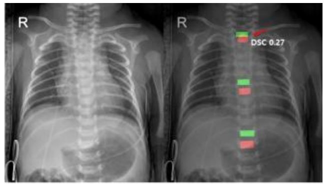


(B)

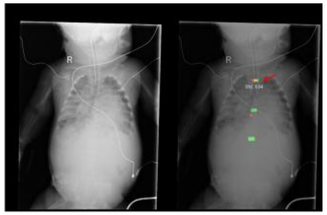


(C)

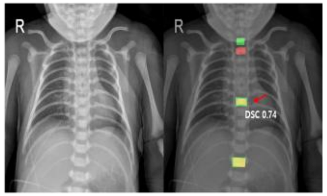


(D)

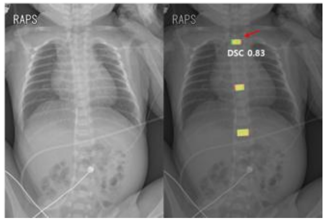


**Supplementary Figure 3. Representative segmentation results at different DSC thresholds (0.3–0.8).**

**Image pairs show the original (left) and overlay (right) images. Visual concordance improved as DSC increased and supported the use of DSC ≥0.5 as a clinically acceptable cutoff.**

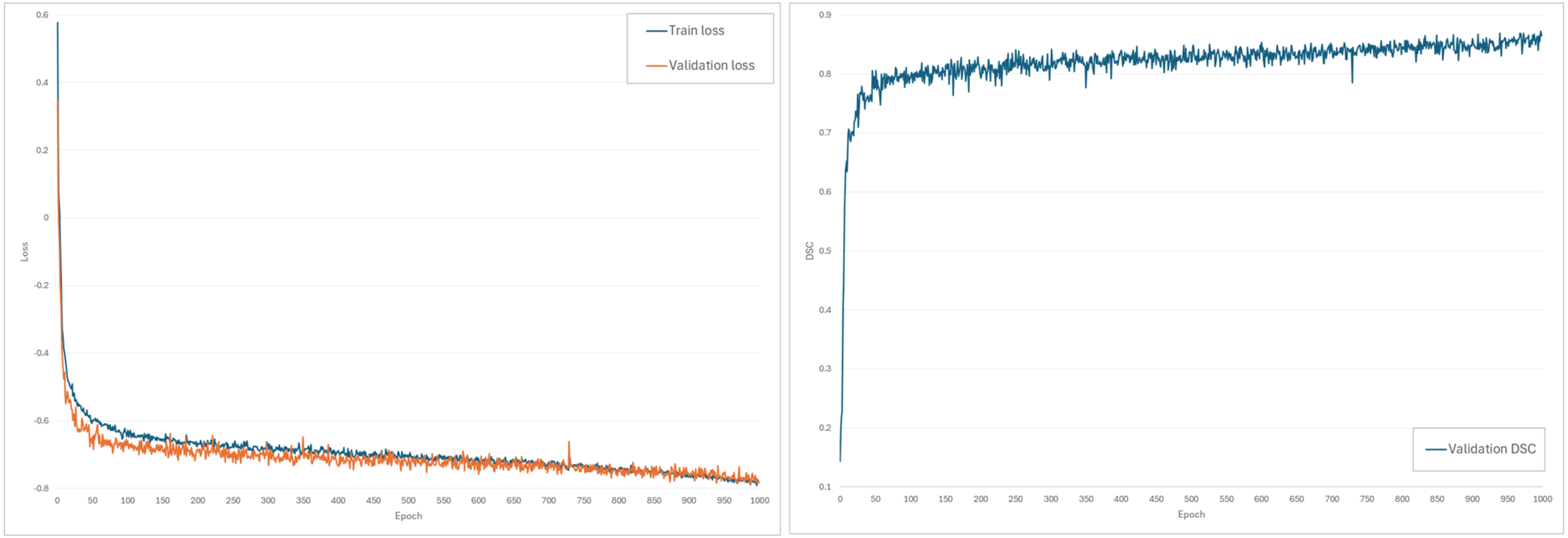


**(A)** **(B)**

**Supplementary Figure 4. Learning curves of the nnU-Net segmentation model during training.**

**(A) Training and validation loss curves over 1,000 epochs, showing stable convergence with no signs of overfitting.**

**(B) Validation Dice Similarity Coefficient (DSC) curve demonstrating performance saturation at around 0.80-0.85.**
